# Electrocardiograms for cardiomyopathy risk stratification in children with anthracycline exposure

**DOI:** 10.1186/s40959-019-0045-6

**Published:** 2019-08-07

**Authors:** Lajja Desai, Lauren Balmert, Jennifer Reichek, Amanda Hauck, Katheryn Gambetta, Gregory Webster

**Affiliations:** 1 Division of Cardiology, Ann and Robert H. Lurie Children’s Hospital of Chicago, 225 East Chicago Avenue, Box 21, Chicago, IL 60611 USA; 2 Northwestern University Feinberg School of Medicine, 420 East Superior Street, Chicago, IL 60611 USA; 3 Division of Hematology/Oncology, Ann and Robert H. Lurie Children’s Hospital of Chicago, 225 East Chicago Avenue, Box 30, Chicago, IL 60611 USA

**Keywords:** Pediatric oncology, Cardiomyopathy, Electrocardiograms, Anthracyclines

## Abstract

**Background:**

Early recognition of anthracycline-induced cardiomyopathy may reduce morbidity and mortality in children, but risk stratification tools are lacking. This study evaluates whether electrocardiogram (ECG) changes precede echocardiographic abnormalities in children with anthracycline-induced cardiomyopathy.

**Methods:**

We performed a retrospective analysis of 589 pediatric cancer patients who received anthracyclines at a tertiary referral center. ECG endpoints were sum of absolute QRS amplitudes in the 6 limb leads (ΣQRS(6 L)) and corrected QT interval (QTc). Cardiomyopathy was defined by echocardiogram as ejection fraction < 50%, shortening fraction < 26%, or left ventricular end-diastolic diameter z-score > 2.5.

**Results:**

Median age at start of therapy was 7.8 years (IQR 3.7–13.6); median follow-up time was 3.6 years (IQR 1.1–5.8). 19.5% of patients met criteria for cardiomyopathy. Male sex, race, older age at first dose, and larger body surface area were associated with development of cardiomyopathy. A 0.6 mV decrease in ΣQRS(6 L) and 10 ms increase in QTc were associated with an increased risk of developing cardiomyopathy with hazard ratios of 1.174 (95% CI = 1.057–1.304, *p* = 0.003) and 1.098 (95%CI = 1.027–1.173, *p* = 0.006) respectively. Kaplan-Meier estimates showed a lower chance of cardiomyopathy-free survival for QTc ≥ 440 ms and ΣQRS(6 L) ≤ 3.2 mV over time. After controlling for confounders, total anthracycline dose predicted a decrease in ΣQRS(6 L) and an increase in QTc independent of cardiomyopathy status (*p* = 0.01 and *p* < 0.001 respectively). Cardiotoxic radiation did not predict changes in ECG parameters. Cardiomyopathy was associated with increased mortality (34% versus 12%, *p* < 0.001).

**Conclusion:**

In children receiving anthracyclines, decrease in ΣQRS(6 L) and QTc prolongation are associated with increased risk of developing cardiomyopathy. ECG is a potential non-invasive risk stratification tool for prediction of anthracycline-induced cardiomyopathy and requires prospective validation.

## Background

Anthracyclines are an important component of chemotherapy in pediatrics; however, they carry cardiotoxic risk [1]. Prevention or early recognition of anthracycline-induced cardiomyopathy is important in children to reduce long-term morbidity and mortality. Anthracycline-induced cardiomyopathy can develop acutely (within the first week of drug exposure), early (within one year of drug exposure) or late (greater than one year after drug exposure). Early toxicity may be reversed with discontinuation of the drug, which makes timely recognition of cardiomyopathy especially crucial. Previously studied risk factors for anthracycline-induced cardiomyopathy include cumulative dose of anthracyclines received, rate of exposure, concomitant exposure to cranial or mediastinal radiation, female sex, age at drug exposure, black race, and genetic predisposition [1–6].

Although there are long-term cardiotoxicity screening guidelines for survivors of childhood, adolescent and young adult cancers developed by The Children’s Oncology Group (COG), there continues to be debate on the diagnostic modalities to use and frequency of monitoring. Additionally, there are no current guidelines on the timing or type of cardiac testing outside of echocardiograms that should be done for children during anthracycline treatment and in the acute and early exposure period [1–3, 7]. Myocardial damage may occur before echocardiographic changes, making it important to investigate potential predictive testing. Electrocardiograms (ECGs) are a core diagnostic testing modality in cardiac care and expert interpretation is readily available. Additionally, ECGs are inexpensive and technically straightforward to obtain. Preliminary studies have evaluated ECG changes after anthracycline exposure; however, these changes have not been extensively studied in larger or primarily pediatric cohorts [1, 8–11].

This study aims to evaluate ECG findings in children at a tertiary care institution who have received anthracycline therapy and determine if ECG changes precede echocardiographic findings of cardiomyopathy. This may help answer whether ECGs can serve as a non-invasive tool to screen for cardiomyopathy during and after anthracycline therapy.

## Methods

### Characteristics of participants and inclusion/exclusion criteria

This was a retrospective cohort study at a tertiary care pediatric referral center using electronic medical records and the institution’s oncology pharmacy database. Institutional review board approval was obtained in this HIPAA-compliant study. Informed consent was not required. All pediatric patients from 2006 to 2014 who received anthracyclines as part of their chemotherapy regimen were included in the study population. Echocardiograms were typically obtained by the patient’s oncology team based on COG guidelines unless additional testing was deemed necessary based on the patient’s clinical status. Other cardiac testing was obtained by the patient’s care team based on clinical judgement, so patients did not undergo a pre-specified ECG protocol. Patients without an ECG or echocardiogram were excluded from analysis.

### ECG evaluation

All ECGs were recorded and analyzed on a digital system (MUSE SP2 v8.0, General Electric Corporation). ECGs were digitized at 500 Hz, and measurements were verified manually by a single investigator (LD) blinded to knowledge of the echocardiographic findings. Two ECG endpoints were pre-specified: the sum of absolute QRS amplitudes in the 6 limb leads (ΣQRS(6 L)) and the corrected QT (QTc) interval. These endpoints were chosen based on preliminary data from previously published work [8, 9, 11]. Amplitudes were measured in millivolts (mV) from peak to trough of the QRS. The QTc was measured in milliseconds (ms) using Bazett’s formula and standard T-wave tangent methodology [12].

### Echocardiogram evaluation

Due to lack of clear consensus on echocardiographic parameters for definition of cardiomyopathy, cardiomyopathy was defined based on clinically actionable thresholds both at our institution and in the literature [7, 13–17]. Patients met criteria for cardiomyopathy by either having systolic dysfunction or a dilated left ventricle. Systolic dysfunction was defined by either an ejection fraction (EF) less than 50% or a shortening fraction (SF) less than 26%. A dilated left ventricle was defined by a left ventricular end diastolic dimension (LVEDD) z-score greater than 2.5. EF of < 50% was chosen as this value necessitates echocardiographic reassessment, consideration of chemotherapy change, and possible heart failure treatment [16]. SF of < 26% was based on its correlation to an EF of approximately 50% with m-mode extrapolation and the pediatric oncology standards for change in anthracycline therapy at our institution. LVEDD z-score > 2.5 was included in our analysis since dilated cardiomyopathy can also occur secondary to anthracycline therapy and may require intervention and/or treatment modification [18].

### Statistical analysis

Patient characteristics were summarized using descriptive statistics including mean, standard deviations, median values and interquartile ranges as appropriate. Categorical variables were summarized using counts and percentages. Comparisons between groups by cardiomyopathy status were based on either two-sample t-tests or Wilcoxon’s rank sum test (for continuous variables) and the Chi-square or Fisher’s exact test (for categorical variables). A two-sided *p*-value < 0.05 was considered statistically significant, and no adjustments for multiplicity were made.

Cox regression models with time-dependent covariates explored associations between ECG measures and time from first ECG to development of cardiomyopathy. Subjects were included if they had at least one ECG prior to the echocardiogram identifying cardiomyopathy, and for censored subjects at least one ECG prior to the last negative echocardiogram. A 0.6 mV change in ΣQRS(6 L) was chosen as the initial unit for evaluation because each vertical box represents 0.1 mV on a standard ECG, and an average amplitude change of less than 1 box per lead is difficult to measure in clinical practice. A 10 ms change in QTc interval was chosen as the initial unit for evaluation because it is typically the minimum clinically relevant interval.

Receiver operating characteristic curves of QTc and ΣQRS (6 L) values on the ECGs prior to the echocardiograms showing cardiomyopathy were created to determine Youden’s cutoff for each measure - the point where sensitivity and specificity of the measure is maximized. This cutoff was determined to be 440 ms for the QTc (PPV = 0.273, NPV 0.857) and 3.2 mV (PPV = 0.268, NPV 0.849) for the ΣQRS (6 L). These cutoffs were then used for Kaplan-Meier estimates to demonstrate the probability of developing cardiomyopathy in patients stratified by QTc intervals > 440 ms and < 440 ms and ΣQRS (6 L) ≤ 3.2 mV and > 3.2 mV (on ECGs prior to development of cardiomyopathy or last negative echocardiogram) both from the time of ECG and from the time of therapy completion.

Differences in ΣQRS(6 L) and QTc interval between ECGs before and after anthracycline therapy were compared for the subset of patients with available data. Multiple linear regression models were used to assess the effect of anthracycline dose and cardiotoxic radiation on post-therapy ECG measures adjusting for potential confounders including sex, age at first dose, body surface area (BSA), race (white, black, Hispanic, other), time between pre- and post-therapy ECGs, and pre-therapy ECG measurements. Cardiotoxic radiation was calculated as the total radiation dose a patient received to body fields that are reported to increase the risk of cardiotoxicity by COG [12]. Lastly, changes in ECG measures by cardiomyopathy status were compared using independent two-sample t-tests. In this analysis, post-treatment ECG was defined as the closest ECG on or after development of cardiomyopathy or the closest ECG on or after the last available echocardiogram in patients without cardiomyopathy. Patients were excluded if they did not have an ECG meeting this post-treatment definition or if their first ECG was after development of cardiomyopathy.

## Results

### Overall cohort

**Fig. 1:**
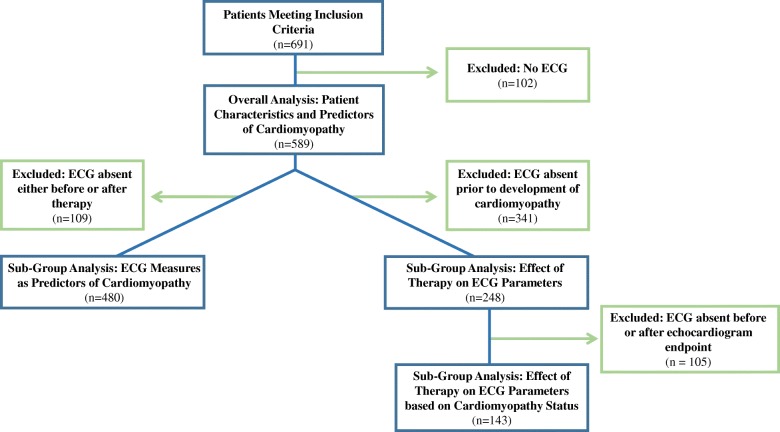
Diagram outlining the number of patients included for each analysis

There were 691 eligible pediatric patients in the study period, of which 102 patients were removed because an ECG was not available. The flow diagram in Fig. 1 provides details on the number of patients included for each analysis.

### Patient characteristics and predictors of cardiomyopathy

The median age at the start of anthracycline therapy was 7.8 years (IQR 3.7–13.6). The median follow-up time after completion of anthracycline therapy to last ECG or echocardiogram was 3.6 years (IQR 1.1–5.8). There were 115 patients (19.5%) who met criteria for cardiomyopathy. Male sex, race, older age at first dose, larger BSA and total anthracycline dose were associated with development of cardiomyopathy (Table 1). Cardiomyopathy was associated with increased mortality (34% versus 12%, *p* < 0.001).

**Table 1:** Patient demographics and treatment characteristics by cardiomyopathy status^a^

	Cardiomyopathy (*N* = 115, 19.5%)	No Cardiomyopathy (*N* = 474, 80.5%)	*P*-value
Sex			
Male	73 (63.5)	249 (52.5)	0.03
Female	42 (36.5)	225 (47.5)	
Age at First Dose (years)	10.8 (4.9–15.1)	7.1 (3.4–12.9)	< 0.001
Race			
White	57 (50.4)	244 (51.6)	0.03
Hispanic	32 (28.3)	160 (33.8)	
Black	18 (15.9)	34 (7.2)	
Other (including Asian)	6 (5.3)	35 (7.4)	
Unknown	2 (1.7)	1 (0.2)	
Body Surface Area (m^2^)	1.20 (0.74–1.66)	0.88 (0.62–1.42)	< 0.001
Diagnosis			
Leukemia	57 (49.6)	253 (53.4)	0.08
Solid Tumors	36 (31.3)	126 (26.6)	
Lymphoma	17 (14.8)	82 (17.3)	
Brain Tumors	1 (0.9)	10 (2.1)	
Multiple Diagnoses	4 (3.5)	3 (0.6)	
Anthracycline Dose Total (mg/m^2^)	236.0 (153.0–329.0)	164.5 (92.0–232.0)	< 0.001
Cardiotoxic Radiation			
Yes	42 (36.5)	152 (32.1)	0.36
No	73 (63.5)	322 (55.7)	
Vital Status			
Alive	76 (66.1)	417 (88.0)	< 0.001
Died	39 (33.9)	57 (12.0)	

^a^Data presented as number of patients(%) or median (interquartile range)

More patients met criteria for cardiomyopathy based on systolic dysfunction parameters versus left ventricular dilation (79% versus 19%) with 2% meeting criteria for both. The median time to development of cardiomyopathy after initiation of anthracycline therapy was 0.1 years (0–0.7). A majority of patients developed cardiomyopathy in the acute/early period of ≤ 1 year after start of therapy (*N* = 87/115, 76%). Among the 87 patients who developed cardiomyopathy in the acute/early period, 25 patients developed cardiomyopathy before the last dose of anthracycline was administered.

### Sub-group analysis: ECG measures as predictors of cardiomyopathy (*n* = 480/589)

**Fig. 2:**
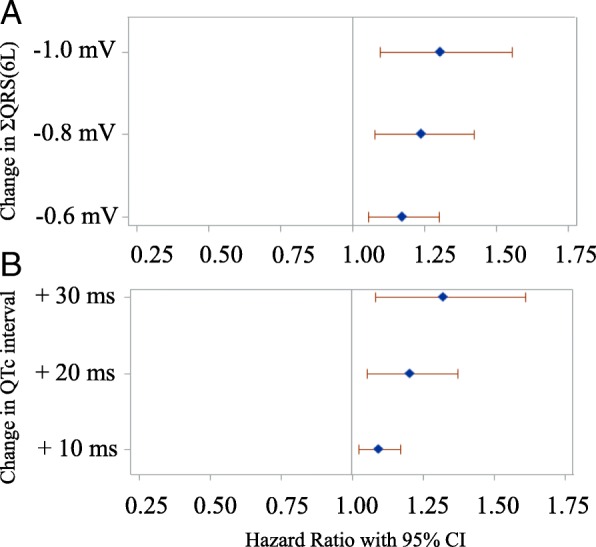
Forest plots show hazard ratios with 95% confidence intervals of the risk of developing cardiomyopathy after anthracycline therapy for a range of ΣQRS(6 L) and QTc measures. **a**: The hazard ratio increases as the ΣQRS(6 L) decreases. **b**: The hazard ratio increases as the QTc interval increases

The median number of ECGs prior to the echocardiogram identifying cardiomyopathy or the last negative echocardiogram was 2 with a range of 1 to 50 ECGs. This median value was consistent between both cohorts with and without cardiomyopathy. Cox models with time-dependent ECG predictors identified a hazard ratio of 1.174 (17% increased risk of developing cardiomyopathy, *p* = 0.003) for a 0.6 mv decrease in ΣQRS(6 L) and a hazard ratio of 1.098 (10% increased risk of developing cardiomyopathy, *p* = 0.006) for a 10 ms increase in QTc interval (Table 2). The forest plots in Fig. 2 show the hazard ratios with 95% confidence intervals for additional ΣQRS(6 L) and QTc values. The hazard ratio increases as the ΣQRS(6 L) decreases and as the QTc interval increases.

**Table 2:** Cox regression models with ECG measures as predictors for time to development of cardiomyopathy (*N* = 480)

Model	Covariate^a^	Unit Change	Hazard Ratio	95% CI	*P*-value
1	ΣQRS (6 L)	0.6 mV	1.174	1.057–1.304	0.003
2	QTc	10 ms	1.098	1.027–1.173	0.006

^a^ECG measures were evaluated as time-dependent covariates

ΣQRS (6 L): sum of QRS amplitude in 6 electrocardiogram limb leads, QTc: corrected QT interval

**Fig. 3:**
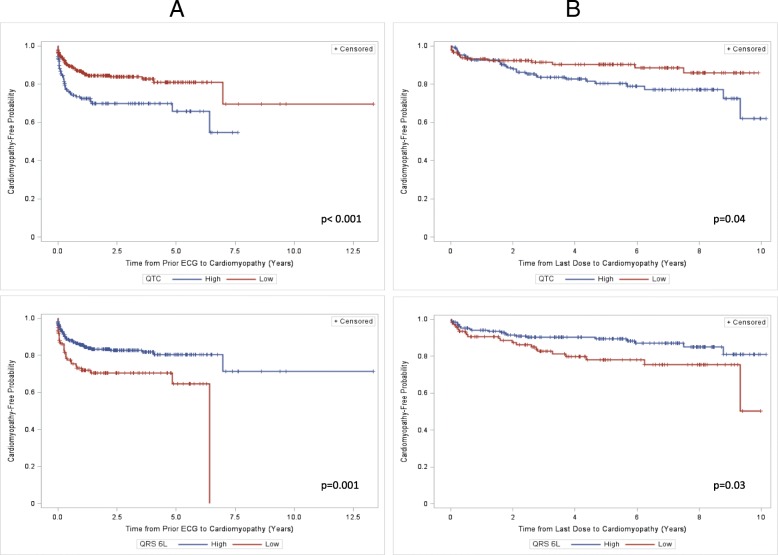
Kaplan-Meier estimates show the probability for patients to remain “cardiomyopathy-free” over time. There is a lower chance of cardiomyopathy-free survival for QTc ≥ 440 ms (“high”) and ΣQRS(6 L) ≤ 3.2 mV (“low”) over time. Time “zero” for column **a** (n=480) is the electrocardiogram (ECG) preceding echocardiographic evidence of cardiomyopathy and for column **b** (n=303) is the last anthracycline treatment dose. The patients in column **b** are the subset from column **a** who had completed chemotherapy at the time of ECG analysis

The median time from the most recent ECG prior to the first indication of cardiomyopathy was 86 days (IQR 8–209), and the median time from the first available ECG to next echocardiogram was 91 days (IQR 19–336). The median time from the start of treatment to the first available ECG was − 1 day (IQR − 34 to 38). The median time from the start of treatment to the next available ECG was 110 days (IQR 31–364). Figure 3 shows the time to development of cardiomyopathy stratified by ECG cutoffs as determined by Youden’s index. The probability that a patient develops cardiomyopathy over time is higher for patients who have an ECG with QTc ≥ 440 ms or ΣQRS (6 L) ≤ 3.2 mV both from the time of ECG and from the time of therapy completion.

### Sub-group analysis: effect of therapy on ECG parameters (*n* = 248/589)

In this sub-group, an unadjusted analysis evaluating all patients with an ECG before and after anthracycline therapy showed a 0.2 mV median decrease in ΣQRS(6 L) (*p* = 0.01) and a 4 ms average increase in QTc interval (*p* = 0.02) after completion of therapy (Table 3). In linear regression models only adjusting for pre-therapy ECG measures, an increase in total anthracycline dose was associated with a decrease in ΣQRS(6 L) and an increase in QTc interval (*p* < 0.001 for both). Multiple linear regression models adjusting for additional confounders including sex, age at first dose, BSA, race, and time between pre- and post-therapy ECG measures showed the total anthracycline dose remained a modest but statistically significant predictor for a decrease in ΣQRS(6 L) and an increase in QTc interval (Table 4).

**Table 3:** Comparison of ECG measures before and after therapy (*N* = 248)

	Before therapy^a^	After therapy^a^	*P*-value
ΣQRS(6 L) (mV)	4.2 (3.2–5.0)	3.9 (3.2–4.9)	0.01
QTc (ms)	427.0 ± 20.9	431.2 ± 25.5	0.02

^a^Data presented as median (interquartile range) or mean ± standard deviation

ΣQRS(6 L): sum of QRS amplitudes in 6 electrocardiogram limb leads, QTc: corrected QT interval

**Table 4:** Multiple linear regression models^a^ showing effect of anthracycline dose on post-therapy ecg measures (*N* = 248)

Model	Outcome	Anthracycline dose estimate (mg/m2)	95% CI	*P*-value
1	ΣQRS (6 L)	– 0.001	– 0.002 to −0.0003	0.01
2	QTc	+ 0.06	+ 0.03 to + 0.09	< 0.001

^a^Adjusted for sex, age at first dose, body surface area, race (white, black, Hispanic, other), time between pre- and post-therapy ECG measures, and pre-therapy ECG measures

ΣQRS(6 L): sum of QRS amplitudes in 6 electrocardiogram limb leads, QTc: corrected QT interval

Cardiotoxic radiation was delivered as adjunct therapy in 194/589 or 33% of patients. Total dose of radiation was not associated with changes in post-therapy ΣQRS(6 L) or the QTc interval. This was true in both simple and multiple linear regression models and was also true when radiation was stratified as a binary variable of radiation exposure versus no radiation exposure.

### Sub-group analysis: effect of therapy on ECG parameters based on cardiomyopathy status

#### (*n* = 143/589)

There were 62 patients (25%) in this sub-group who met criteria for cardiomyopathy with 11 patients developing cardiomyopathy before completing therapy. When comparing the change in ECG parameters before and after anthracycline therapy in the cardiomyopathy versus no cardiomyopathy group, there was a greater decrease in ΣQRS(6 L) in the cardiomyopathy group (Table 5). Although there was no statistically significant difference in the QTc interval change between these groups, the data trended in the expected direction with a greater increase on average in patients who developed cardiomyopathy (Table 5).

**Table 5:** Pre- and post-treatment ECG changes in patients by cardiomyopathy status (N = 143)

	Cardiomyopathy^a^ (*N* = 53)	No Cardiomyopathy^a^ (*N* = 90)	*P*-value
Change in ΣQRS (6 L)	– 0.6 ± 1.0	– 0.2 ± 1.1	0.02
Change in QTc	7.5 ± 35.5	4.6 ± 31.5	0.61

^a^Data presented as mean ± standard deviation

ΣQRS(6 L): sum of QRS amplitudes in 6 electrocardiogram limb leads, QTc: corrected QT interval

## Discussion

**Fig. 4:**
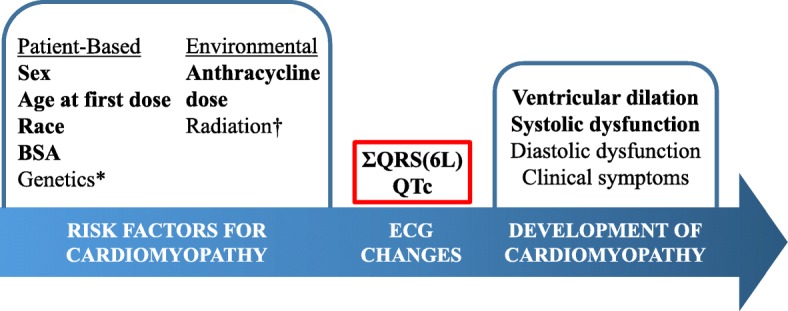
Multiple factors increase a patient’s risk of anthracycline-induced cardiomyopathy in children. ECGs may identify these patients before echocardiographic and clinical changes. *Genetics was not evaluated in this study. †Cardiotoxic radiation has been previously described as a risk factor but did not have a statistical association with cardiomyopathy in this study

Our study demonstrates that clinically measurable ECG changes, specifically a decrease in ΣQRS(6 L) and prolongation of the QTc interval, are associated with an increased risk of developing anthracycline-induced cardiomyopathy. As survival rates have improved in pediatric oncology, focus has shifted on reducing associated morbidities like cardiotoxicity to ensure long-term quality of life [19]. Reduction, recognition and treatment of these morbidities via personalized cancer therapy requires consideration of baseline patient factors, identification of risk stratification tools during and after therapy, and clear definitions of the adverse outcome. Figure 4 shows a representation of this process for pediatric anthracycline-induced cardiomyopathy. Supported by our study and prior literature, multiple baseline biologic (sex, gender, age, race, BSA, genetics) and environmental (anthracycline dose, radiation) factors can contribute to a patient’s risk of developing cardiomyopathy [1–6]. Cardiomyopathy is typically recognized via echocardiographic and/or clinical manifestations. Our study focuses on the period of surveillance after treatment is initiated to identify a potential screening tool for the cardiomyopathy endpoint. Based on our findings, ECGs are an inexpensive and readily available tool that may aid in evaluation of individual patient risk during and after therapy. ECGs should be considered as part of comprehensive cardio-oncology surveillance, but a specific protocol for screening cannot be established without prospective study design.

Existing literature has attempted to show a relationship between ECG measures after anthracycline therapy and echocardiographic findings. An initial study analyzed ECGs for pediatric patients treated with Adriamycin at specified intervals after completion of chemotherapy: 2 months, 1 year, and 3 years [20]. Although a higher rate of ventricular dysfunction was observed in patients with a prolonged QTc, the difference was not statistically significant. This is likely due to an underpowered study number with only 43 patients at 3 years of follow-up. A small adult study with 26 patients did show an increase in QTc interval and decrease in QRS voltages after anthracycline treatment and suggested an association to LV dysfunction on echocardiogram; however, this study was also underpowered and had short follow-up times [9].

A more recent analysis by Markman et al. attempted a similar approach to our study to determine if an ECG finding, specifically the QTc interval, precedes development of left ventricular dysfunction defined by shortening fraction [8]. A Kaplan-Meier estimate showed a higher cumulative rate of left ventricular dysfunction in patients who had a maximum QTc > 450 ms at any point during the study period, including after the development of cardiomyopathy. The definition of left ventricular dysfunction was unclear in this second analysis. Although this study had more patients than prior studies, authors suggested they had limited numbers for sufficient power, and their study population only involved patients > 18 years of age.

Our study expands on the concept presented by prior literature by analyzing a larger patient cohort, including a more consistent and comprehensive definition of cardiomyopathy, evaluating an additional ECG parameter of ΣQRS (6 L), determining cutoffs for QTc and ΣQRS (6 L) using a receiver operating characteristic curve, and using only ECG values that precede development of cardiomyopathy for analysis. Additionally, we show how changes to ECG measures over time affect the risk of developing cardiomyopathy.

Anthracycline therapy has been shown to prolong the QTc interval in prior small studies in adult survivors of either pediatric or adult malignancies [8, 9, 11]. A slightly larger pediatric study with 100 patients also showed QTc prolongation after anthracycline therapy [10]. Decreases in QRS voltages have been associated with left ventricular dysfunction on echocardiogram both after anthracycline use and in patients with heart failure due to other causes [9, 21]. While the total anthracycline dose was shown to decrease the ΣQRS(6 L) and prolong the QTc interval in our study, the data suggest the magnitude of ECG changes due to development of cardiomyopathy were larger than changes due to anthracycline exposure alone. In addition, cardiotoxic radiation therapy did not alter the ΣQRS(6 L) or QTc interval, suggesting the ECG changes in patients exposed to anthracyclines should not be dismissed as secondary to therapeutic radiation exposure. A prospective long-term study should be considered to reproduce our results using a systematic approach, and we established ΣQRS(6 L) and QTc as two appropriate outcome measures for evaluation.

A large percentage of patients in this study met echocardiographic criteria for cardiomyopathy in the acute/early period (76%, *n* = 87) with some patients even developing toxicity before the last dose of an anthracycline was given (*n* = 25/87). The patients who developed cardiomyopathy before their last dose of anthracyclines may have the most potential to benefit from ECG-based risk stratification, since heart failure therapy could be initiated and treatment protocols modified before echocardiographic changes are noted [22]. As development of cardiomyopathy was associated with mortality, this further supports early incorporation of ECG surveillance.

There are limitations to this study, particularly due to its retrospective nature. Each patient’s clinical team determined when to obtain ECGs since a specific guideline is not currently available. This could potentially create bias in the study as patients with clinical concern many have more data points for analysis and could have extrinsic causes for ECG findings related to their complex oncologic care (i.e. electrolyte disturbance). This was somewhat mitigated by our inclusion of both patients with and without cardiomyopathy in the Cox and Kaplan-Meier analyses with a consistent median number of ECGs for patients with and without development of cardiomyopathy. Additionally, dichotomization of the primary predictor variable (presence or absence of cardiomyopathy) can be a subjective process, especially when performed using only echocardiographic means without clinical correlation. There is suggestion in small pediatric studies that diastolic dysfunction can occur as part of anthracycline related cardiotoxicity even at lower anthracycline doses [23, 24]. Diastolic dysfunction was not studied as an outcome variable in this study due to inconsistent availability of diastolic interpretation. Modern imaging modalities and techniques such as 3D echocardiography, strain and magnetic resonance imaging may be useful to detect early cardiotoxicity in these patients; however this data was also not consistently available for our review during the available study period. Finally, our study had variable follow-up periods for each patient and could not assess all late-onset cardiomyopathy patients. This may affect interpretation of the predictors/risk factors of cardiotoxicity. For example, female sex has been shown in prior literature to be a predictor for late development of cardiomyopathy [4]. In our study, male sex was associated with the development of cardiomyopathy, possibly due to the shorter available follow-up period and the cohort being restricted to pediatrics. Additionally, prior literature has shown younger age at diagnosis to be a predictor for development of cardiomyopathy [1]. Although our study contradicts the prior literature and finds that older age at diagnosis increases the risk of cardiomyopathy, this may again be due to the tighter age range of our study population and limited available follow-up time.

## Conclusion

A decrease in ΣQRS(6 L) or prolongation of the QTc interval increases the risk of developing cardiomyopathy in pediatric patients receiving anthracycline therapy. Higher total anthracycline doses were associated with decreases in the ΣQRS(6 L) and prolongation of the QTc interval. The decrease in ΣQRS(6 L) after therapy was greater in patients who developed cardiomyopathy. Cardiotoxic radiation was not associated with changes in the ECG parameters measured. ECGs are a potential non-invasive, inexpensive tool for prediction of anthracycline-induced cardiomyopathy and require prospective validation. Incorporation of ECG evaluation in cardio-oncology monitoring regimens in pediatric patients should be carefully considered.

## Data Availability

The datasets used and/or analyzed during the current study are available from the corresponding author on reasonable request.

## Abbreviations

BSA: body surface area
COG: Children’s Oncology Group
ECG: Electrocardiogram
EF: Ejection fraction
LVEDD: Left ventricular end diastolic dimension
ms: millisecond
mV: millivolt
QTc: corrected QTc interval
SF: Shortening fraction
ΣQRS(6 L): sum of absolute QRS amplitudes in the 6 limb leads
